# Deleterious protein-coding variants in diverse cattle breeds of the world

**DOI:** 10.1186/s12711-021-00674-7

**Published:** 2021-10-15

**Authors:** Sankar Subramanian

**Affiliations:** GeneCology Research Centre, School of Science, Technology and Engineering, The University of the Sunshine Coast, 1 Moreton Parade, QLD 4502 Petrie, Australia

## Abstract

The domestication of wild animals has resulted in a reduction in effective population sizes, which can affect the deleterious mutation load of domesticated breeds. In addition, artificial selection contributes to the accumulation of deleterious mutations because of an increased rate of inbreeding among domesticated animals. Since founder population sizes and artificial selection differ between cattle breeds, their deleterious mutation load can vary. We investigated this question by using whole-genome data from 432 animals belonging to 54 worldwide cattle breeds. Our analysis revealed a negative correlation between genomic heterozygosity and nonsynonymous-to-silent diversity ratio, which suggests a higher proportion of single nucleotide variants (SNVs) affecting proteins in low-diversity breeds. Our results also showed that low-diversity breeds had a larger number of high-frequency (derived allele frequency (DAF) > 0.51) deleterious SNVs than high-diversity breeds. An opposite trend was observed for the low-frequency (DAF ≤ 0.51) deleterious SNVs. Overall, the number of high-frequency deleterious SNVs was larger in the genomes of taurine cattle breeds than of indicine breeds, whereas the number of low-frequency deleterious SNVs was larger in the genomes of indicine cattle than in those of taurine cattle. Furthermore, we observed significant variation in the counts of deleterious SNVs within taurine breeds. The variations in deleterious mutation load between taurine and indicine breeds could be attributed to the population sizes of the wild progenitors before domestication, whereas the variations observed within taurine breeds could be due to differences in inbreeding level, strength of artificial selection, and/or founding population size. Our findings imply that the incidence of genetic diseases can vary between cattle breeds.

**Supplementary Information:**

The online version contains supplementary material available at 10.1186/s12711-021-00674-7.

## Background

Population genetics theories predict that, at low frequencies, deleterious single nucleotide variants (SNVs) can contribute significantly to the heterozygosity of a population [1, 2]. In contrast, SNVs are prevented from reaching high frequencies and are eventually eliminated by purifying selection [2]. Domestication of wild plants and animals results in a population bottleneck because only a small subset of the wild population is sampled to form the founder stock [3]. Artificial selection for desired traits and inbreeding also lead to a further reduction in the effective population sizes during breed formation [4]. As a result, domesticated plants and animals are expected to accumulate an excess of deleterious mutations compared to their wild types. A number of previous studies investigated this issue by comparing the deleterious mutational loads of wild and domesticated plants and animals, and the diversity ratio ($\omega$) between amino acid changing (nonsynonymous) and silent (synonymous) SNVs was used as a measure of deleterious mutational load [3, 5–12]. A previous study [5] showed that *ω* was much higher in domesticated pig breeds than in wild pigs and also much higher in commercial white layer chickens than in wild African village chickens (putatively close to the jungle fowl) [5]. Similarly, much higher *ω* have been reported for domesticated breeds of horse [6], dog [7], rabbit [8], and silkworm [8] compared to their wild relatives, and for cultivated crops such as rice [3, 9], soybean [10], cassava [11], and sunflower [12] compared to their wild progenitors.

All the genetic variants that reduce the fitness of an organism are collectively designated as deleterious mutations. While lethal and highly deleterious variants are immediately removed from the populations, the mildly deleterious variants segregate within a population for a short period of time. In this study, mildly harmful variants (i.e. that reduce fitness) are referred to as deleterious mutations. Natural selection prevents such deleterious mutations from reaching high frequencies and eventually eliminates them from the population [2]. Therefore, deleterious mutations are expected to be present at a low frequency, predominantly in the heterozygous state. However, when the size of a population declines, deleterious mutations drift to high frequencies [1]. Furthermore, a reduction in population size or bottleneck will also increase the number of homozygous deleterious mutations, which result in a deviation from the Hardy–Weinberg equilibrium. For instance, human migration out of Africa resulted in a series of bottlenecks in the non-African populations as they were successively subsampled along the migratory routes, which led to a higher proportion of high-frequency and homozygous deleterious SNVs in non-African compared to African populations [13, 14]. Since the process of domestication also introduces bottlenecks, a similar pattern is expected in domesticated animals. This was confirmed by a previous study that compared the exomes between dog breeds and wild wolves and found a higher proportion of homozygous deleterious SNVs in dogs than in wolves [7]. Similarly, domesticated yak populations were reported to have a larger number of homozygous deleterious amino acid-changing SNVs than that estimated for wild yaks [15]. High-frequency deleterious variants that cause diseases such as retinal degeneration in European cattle breeds have been attributed to result from the process of domestication and artificial selection [16].

Cattle breeds belong to the subspecies *Bos taurus taurus* and/or *Bos taurus indicus* [17]. The level of heterozygosity and the deleterious mutational load in each breed can be determined by the size of their progenitor population [18]. In addition, the differences in the degree and patterns of artificial selection and in the rate of inbreeding can also contribute to the variation in deleterious mutation load among cattle breeds [4]. In this study, we estimated the deleterious mutational load in various cattle breeds and investigated the potential contributions of the above-mentioned factors by analysing whole-genome data from 432 animals belonging to 54 distinct worldwide cattle breeds.

## Methods

### Genome data

Whole-genome data from 108 cows and 314 bulls were obtained from the Bovine Genome Variation Database (BGVD) [19]. These animals belong to 54 breeds, including breeds from Europe (Central and Western Europe), Northeast Asia (Japan and South Korea), Africa (Western Africa and Guinea), Middle East (Iran), South Asia (India, Pakistan, and Sri Lanka) and East Asia (China). The number of individuals in each breed ranged from 1 to 45 [for details, (see Additional file 1: Table S1)]. In this study, indicine × taurus breeds were referred to as indicine due to their similarity in diversity and deleterious mutational load with indicine breeds. To orient the direction of mutations and to find the derived SNVs, we used whole-genome data of American Bison (*Bison Bison*). For this purpose, the pairwise whole-genome alignment between the cow and American Bison created with the LASTZ program was downloaded from the Ensembl genome data resource (ftp://ftp.ensembl.org/pub/release-102/maf/ensembl-compara/pairwise_alignments/btau_ars-ucd1.2.v.bbbi_bison_umd1.0.lastz_net.tar.gz). The corresponding nucleotides of the Bison genome was used to determine the orientation of the cattle SNVs. While the Bovine Genome Variation Database is based on the reference Btau 5.0.1 build, it also contains the corresponding chromosomal coordinates for the reference ARS-UCD1.2. Therefore, we used the ARS-UCD1.2 coordinates to detect variants, link annotations, and for genome evolutionary rate profiling (GERP) score information. Furthermore, the two alleles of each SNV in the cow genome were compared with those in the bison genome. For our analysis, we included only the SNVs that had at least one allele matching with that of the bison SNVs, which allowed us to confirm the variants independently of the build that was used to map them.

### Functional annotations

To identify amino acid changing (nonsynonymous) SNVs and silent (synonymous) SNVs, the genome annotation file containing the information on functional consequences was obtained from the Ensembl server (ftp://ftp.ensembl.org/pub/release-102/variation/vcf/bos_taurus/bos_taurus_incl_consequences.vcf.gz). The coding sequences for 15,392 unique reference genes of the cow were downloaded from the GenBank reference genes database. Using the program *codeml* of the software *PAML* [20], the exact numbers of synonymous and nonsynonymous positions were calculated. In order to identify the deleterious amino acid-changing SNVs, the GERP score was used [21]. The GERP score for each chromosomal position of the cow was calculated based on a whole-genome alignment of 90 mammalian genomes, and this data was downloaded from the *Ensembl* server (http://ftp.ensembl.org/pub/current_compara/conservation_scores/90_mammals.gerp_conservation_score/).

### Determination of the deleteriousness of amino acid changing SNVs

A number of studies have used the GERP score to determine the deleteriousness of SNVs [13, 14, 22, 23]. However, Huber et al. [24] and Lawrie et al. [25] have raised concerns about the use of this score [24, 25]. Huber et al. [24] investigated the relationship between the GERP scores and the fitness effects on the organism in terms of selection coefficient ($s$) and effective population size (${N}_{e}$) and showed that while lower GERP scores (< 0) predict neutral mutations that are not under selective constraints (${N}_{{e}^{S}}$> − 1), high GERP scores (> 5.5) accurately predict deleterious mutations that are under high purifying selection (${N}_{{e}^{S}}$< <  − 1), but GERP scores (0–4.5) with moderate values are ambiguous and are not able to distinguish neutral from deleterious mutations. Furthermore, they showed that selection coefficients of the functional elements in the noncoding regions change significantly over time (functional turnover), and hence the statistical power of predicting deleterious single nucleotide polymorphisms (SNPs) in noncoding regions using the GERP method is low. However, high GERP scores (> 5.5) do have the power to detect deleterious SNPs (those under purifying selection) in zero-fold and two-fold degenerate sites of protein-coding genes [24]. Therefore, based on the results of Huber et al. [24], we used a GERP score threshold higher than 5.5 to identify deleterious nonsynonymous SNVs in the above-mentioned sites of coding genes.

### Data analysis

Nucleotide diversity (*π*) per base was estimated using the following equations [26]:

$${\theta }_{T}= \frac{n}{n-1}\sum_{i=1}^{S}2{p}_{i}\left(1-{p}_{i}\right),$$

$$\pi =\frac{{\theta }_{T}}{L},$$

where ${p}_{i}$ is the allele frequency of SNV $i$, $S$ is the total number of SNVs in the whole genome or exome, $n$ is the number of chromosomes sampled and $L$ is the number of sites or bases in the genome, at synonymous or nonsynonymous positions. The ratio ($\omega$) was estimated as:

$$\omega =\frac{{\pi }_{N}}{{\pi }_{S}},$$

where ${\pi }_{N}$ and ${\pi }_{S}$ are nonsynonymous and synonymous nucleotide diversities. Only biallelic SNVs are used in the analysis. To test the significance between mean estimates, we used the Z-test, and to estimate the strength of relationships, we used the Pearson correlation. The nonparametric Spearman correlation also produced similar strengths of correlations.

## Results

**Fig. 1:**
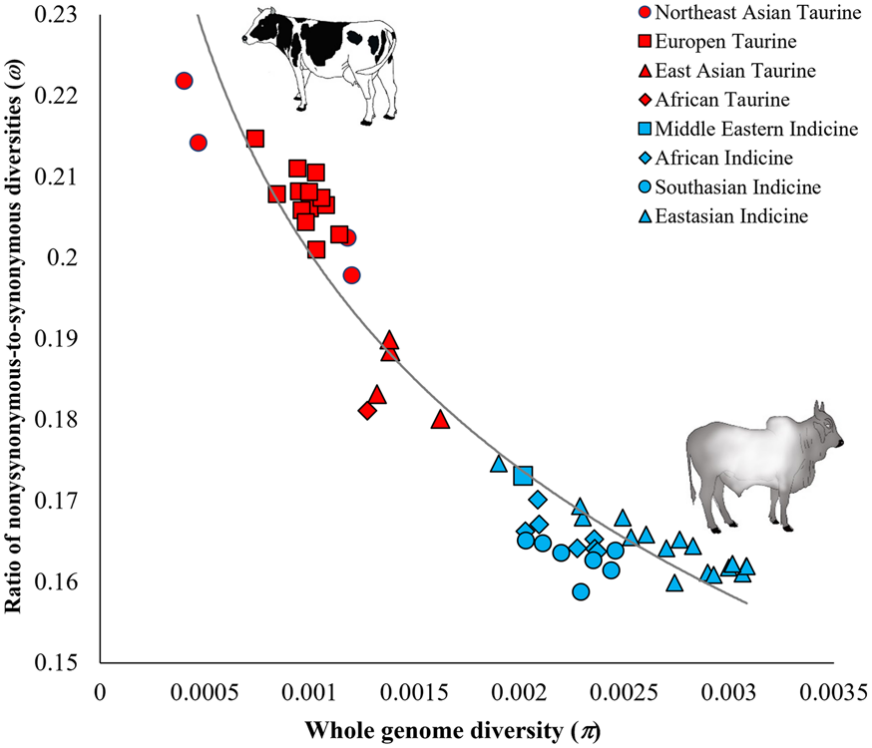
Relationship between whole-genome diversity ($\pi$) and the ratio of nonsynonymous heterozygosity to synonymous heterozygosity ($\omega$) estimated for 54 cattle breeds. Colour codes of the data points distinguish subspecies of the breeds, and shapes denote the geographical locations of the breeds. The correlation was highly significant (Pearson’s correlation *r* = − 0.95, *P* < 0.000001). The best-fitting regression line is shown

To examine the pattern of genomic variation, the whole genome nucleotide diversity was estimated for 54 cattle breeds. The X-axis on Fig. 1 shows that taurine breeds have low diversities (0.0004–0.0016) compared to indicine breeds (0.0020–0.0031). These diversity ranges suggest that, within the taurine breeds (red), there is a four-fold variation in the genome diversity, whereas within the indicine breeds (blue) it does not exceed 70%. The nonsynonymous-to-synonymous diversity ratio ($\omega$) was estimated for each breed, and the values were plotted against the genomic diversities. This analysis revealed a highly significant negative correlation (Pearson *r* = − 0.95, *P* < 0.000001) between the two variables (Fig. 1), which suggests that the breeds with a low genomic diversity have a high proportion of amino acid-changing SNVs compared to those with a high genomic diversity. Hence, taurine breeds have a much higher $\omega$ (0.18–0.22) than the indicine (0.16–0.17) breeds. However, within the taurine breeds, the $\omega$ values observed for European and Northeast Asian (Japanese and Korean) taurine breeds (0.20–0.22) were higher than those for African and East Asian taurine breeds (0.18–0.19). In contrast, *ω* did not vary much among the indicine breeds.

**Fig. 2:**
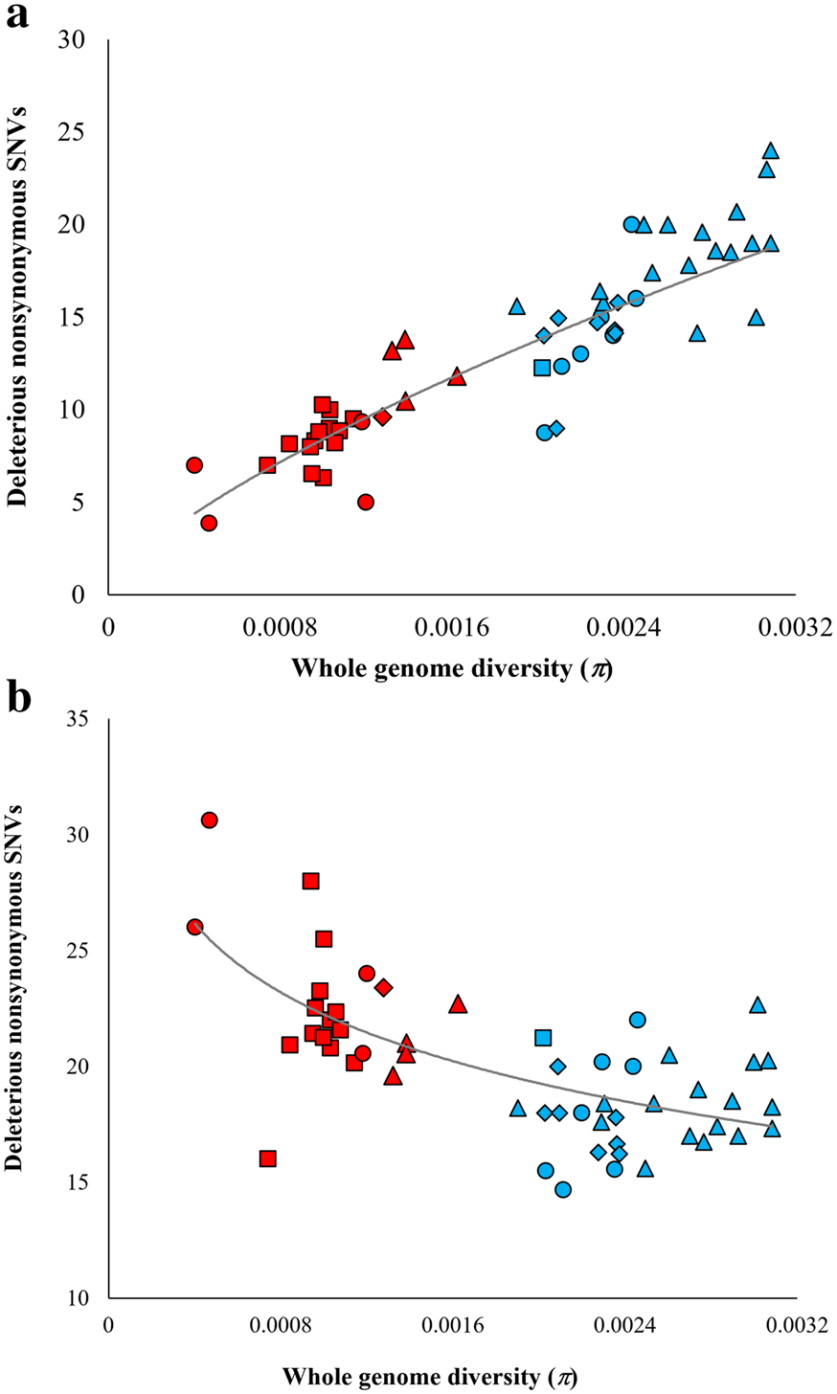
Correlation between whole-genome diversity ($\pi$) and the number of derived deleterious nonsynonymous SNVs per genome. Deleterious SNVs were divided into two groups based on their derived allele frequencies (DAF): **a** low-frequency deleterious SNVs (≤ 0.51) and **b** high-frequency deleterious SNVs (> 0.51). The positive (*r* = − 0.88, *P* < 0.000001) and negative (*r* = − 0.67, *P* < 0.000001) correlations were highly significant and best-fitting regression lines are shown

The $\omega$ estimates indirectly revealed the nonsynonymous deleterious mutation loads in various cattle breeds. We identified the deleterious nonsynonymous SNVs by setting a threshold higher than 5.5 for the GERP score and separated them into two groups: those with a DAF lower than 0.51, and those with a DAF higher than 0.51. The number of derived deleterious amino acid changing SNVs per genome was calculated separately for each DAF category and for each breed. This was done by counting the total number of deleterious low-frequency SNVs observed in a breed and dividing it by the number of animals in the breed. The same was calculated for high-frequency deleterious SNVs. These counts per genome were then correlated with the genomic diversity. This analysis revealed contrasting patterns for high and low-frequency SNVs. Figure 2a reveals a significant positive correlation (*r* = 0.88, *P* < 0.000001) between the number of low-frequency deleterious SNVs per genome and genomic diversity. The number of low-frequency deleterious nonsynonymous SNVs varied drastically between breeds (from 3.9 to 24.0). Analysis of the high-frequency (DAF > 0.51) nonsynonymous deleterious SNVs showed a significant negative relationship (*r* = − 0.67, *P* < 0.000001) between genome diversity and the counts of deleterious SNVs per genome (Fig. 2b). We observed a two-fold difference (from 14.7 to 30.6) in the SNV counts among the breeds. Some of the data points in Fig. 2 represent breeds that comprise only one or two animals, which might influence the results. However, highly significant relationships were also observed when only the breeds with more than two animals were included (*P* < 0.00002).

**Fig. 3:**
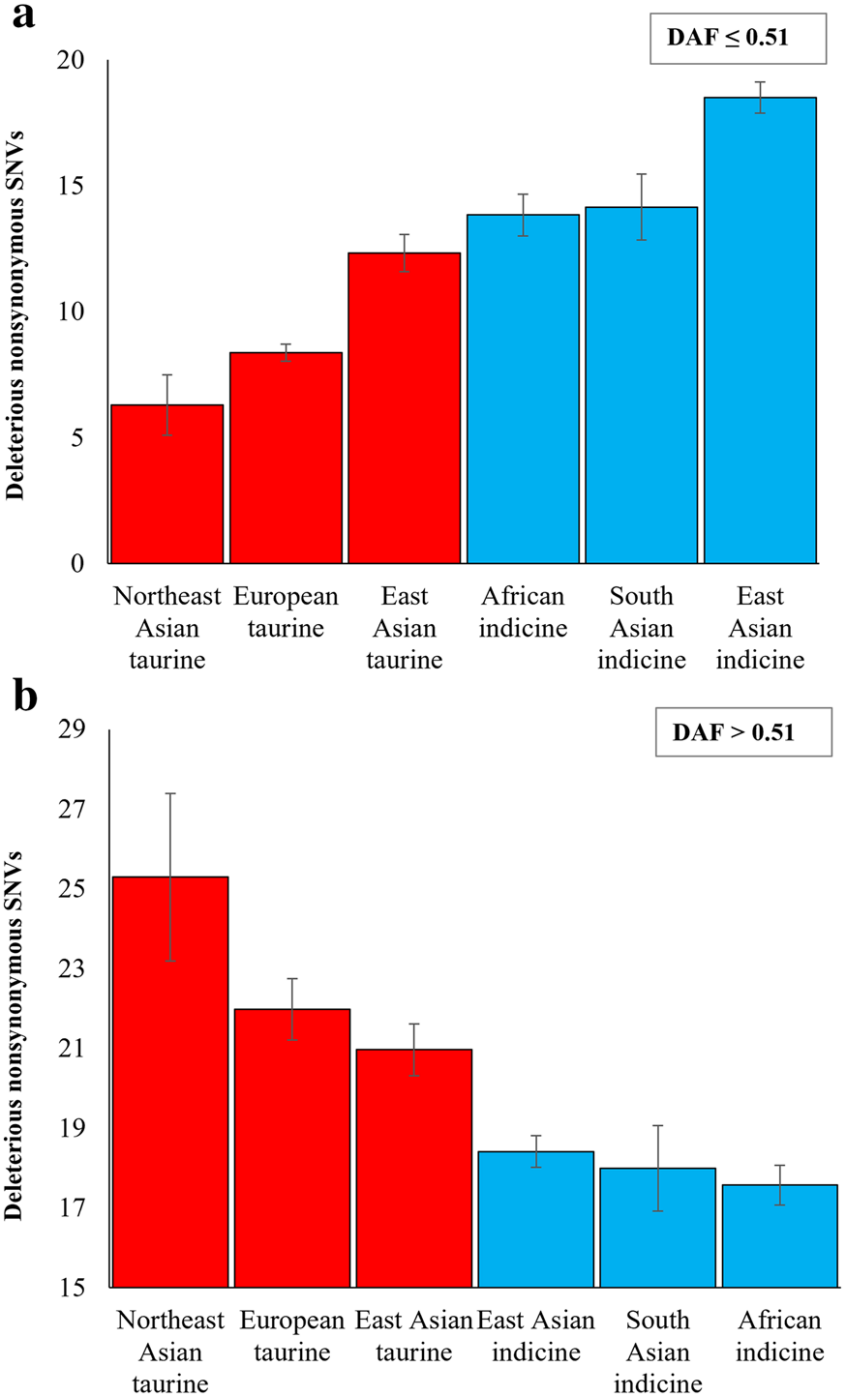
The average number of deleterious derived nonsynonymous SNVs per genome was calculated for each category of cattle breeds that were grouped based on their geographical location and subspecies. **a** Low-frequency SNVs (DAF ≤ 0.51) and **b** high-frequency SNVs (DAF > 0.51). Error bars denote the standard error of the mean

To obtain the pattern of the mutational load in breeds from various geographical locations and belonging to different subspecies, we grouped the breeds into six categories, and the average number of deleterious SNVs per genome for each group was calculated (Fig. 3). On average, the taurine breeds have predominantly high-frequency deleterious SNVs, and the indicine breeds have predominantly low-frequency deleterious SNVs. The number of low-frequency deleterious SNVs varied (between 6.3 and 18.5) between breed categories (Fig. 3a). The average number of low-frequency SNVs present in the indicine breeds was significantly larger than that in the taurine breeds (*P* < 0.00001). Similarly, high-frequency deleterious SNVs also varied between the six groups of breeds (between 17.6 and 25.3) (Fig. 3b). The mean count of high-frequency deleterious SNVs in the taurine breeds was significantly higher than that observed in the indicine breeds (*P* < 0.00001). Within the taurine breeds, the Northeast Asian and European breeds have a larger number of high-frequency deleterious SNVs but a smaller number of low-frequency deleterious SNVs than the East Asian taurine breeds. Such variations were not observed within the indicine breeds.

## Discussion

The whole-genome diversity estimated in this study varied significantly between the cattle breeds analysed and are very similar to previously reported values [27]. The correlation between diversity and $\omega$ suggests a higher nonsynonymous mutation load in breeds with a low diversity than in those with a high diversity. This result is similar to the correlations observed between diversity and $\omega$ estimated for various dog breeds [7] and domestic breeds of rabbit, pig, and chicken [8]. Furthermore, the higher $\omega$ observed for many domestic crop varieties and animal breeds compared to their wild relatives also support our results [3, 5–12, 15, 28]. Previous studies showed that the number of homozygous deleterious SNVs was larger in domesticated canines or yaks than in their respective wild relatives [7, 15]. Since homozygous SNVs represent high-frequency variants, our results are consistent with those reported by the above-mentioned studies.

Previous studies using high-density (HD) SNP arrays revealed a much higher genetic diversity for taurine than for indicine cattle [29–32], e.g. in [29] the observed heterozygosities for indicine breeds were lower than 0.21 and those for taurine breeds were higher than 0.3. However, a deeper analysis detected an ascertainment bias that explained this difference since the SNP array chips were predominantly based on taurine breeds [30, 33, 34]. A number of later studies (including the 1000 bull genomes project) based on whole-genome sequencing data showed that indicine breeds had an almost two-fold higher nucleotide diversity, i.e. ~ 0.003 for indicine versus ~ 0.0015 for taurine breeds [18, 27], which are comparable to our estimates i.e. from 0.0019 to 0.0031 for the indicine versus from 0.0004–0.001 for the taurine breeds. Also, two other studies reported a two-fold larger number of SNPs in indicine than in taurine breeds [35, 36]. Nevertheless, the very low genomic diversity of some of the taurine breeds analysed in our study could also be due to the increased rate of inbreeding resulting from intense selective breeding.

Studies on mitochondrial genomes reported a similar or lower diversity in indicine mitogenomes compared to those of taurine breeds [37–40]. However, this could be attributed to the number and types of haplogroups present in each breed. The haplogroups belonging to the taurine mitochondrial lineage are: T (T1–T5), P, Q, R, and E, and those to the indicine lineage are: I1 and I2 [41, 42]. Unlike the nuclear genome, a single breed population can contain one or many mitochondrial haplogroups, including those from taurine and indicine lineages. Therefore, the mitochondrial genome diversity depends on the combination of haplogroups. Breeds with both indicine (I) and taurine (T, P, Q, R or E) haplogroups have a high diversity, whereas breeds with only indicine or only taurine haplogroups have a low diversity [38–40]. Furthermore, within the taurine lineage, breeds containing multiple haplogroups (e.g. T and P lineages) have a higher diversity than those that have only one haplogroup (e.g. T) [40]. This is because diversity is proportional to the coalescence time (age) of the mitochondrial genomes of the breed [41, 42]. Breeds containing multiple haplogroups are old (longer coalescence time) and their diversity is expected to be high. These patterns are evident from many studies (Table 1 in [40]; Supplementary Table S1 in [39]; and Table 1 in [38]). Therefore, the relationship between breed types (taurine or indicine) and mitochondrial nucleotide diversities is complex and needs to be inferred based on the context of haplogroups. Since the taurine mitochondrial lineage has more (9) haplogroups than the indicine lineage (2), the breeds containing the former lineage tend to have a higher level of diversity than the latter. Due to these uncertainties and confounding factors, the present investigation was restricted to the study of the mutational load of nuclear genomes.

The deleterious SNVs estimated for groups of breeds showed that, on average, taurine cattle breeds have a higher mutational load than indicine breeds. The number of high-frequency deleterious SNVs also varies significantly within the taurine and within the indicine breeds. For instance, Northeast Asian and European taurine breeds have a larger number of high-frequency deleterious SNVs, and a smaller number of low-frequency deleterious SNVs than the East Asian taurine breeds. Population genetic theories predict that breeds (populations) with small effective population sizes are expected to have more high-frequency derived SNVs due to the influence of genetic drift, which is strong in small populations [2, 13]. This expectation also holds true for SNVs with low fitness effects [1]. Therefore, the higher counts of derived deleterious SNVs observed in taurine compared to indicine breeds could be attributed to the difference in the effective population sizes of their progenitors before domestication, as previously suggested [18, 43]. In addition, this could also be due to a difference in the size of the bottleneck that occurred during their respective domestication and breed formation [4]. For instance, East Asian taurine breeds have a larger number of deleterious SNVs than East Asian indicine breeds and this difference may reflect a difference in the effective population sizes of the progenitors of taurine and indicine breeds. However, European and Northeast Asian taurine breeds have much more deleterious SNVs than East Asian taurine breeds, which could be the result of highly selective breeding and a severe bottleneck created by a much smaller number of founders used during the formation of European and Northeast Asian taurine breeds.

## Conclusions

Our study revealed a higher mutation load and a larger number of high-frequency deleterious SNVs in cattle breeds with a low genomic diversity than in those with a high genomic diversity. These results suggest that diversity, deleterious mutation load, and frequency of deleterious mutations are determined by their effective population sizes as predicted by population genetic theories. While we found higher mutational load in taurine breeds than in indicine breeds, mutational load did vary within the taurine breeds owing to differences in their effective population sizes. These results have implications regarding the health of cattle breeds since the mutations causing genetic diseases and their frequencies are expected to vary between breeds. For instance, the incidence of genetic diseases caused by recessive homozygous variations could potentially be higher in breeds that have small effective population sizes.

## Supplementary Information

**Additional file 1: Table S1.** Locations and number of samples for each cattle breed.

## Data Availability

All relevant results are within this paper and its additional file. The whole-genome data used in this study is available at: http://animal.nwsuaf.edu.cn/code/index.php/BosVar.

## References

[1] Crow JK, Kimura M (1970). An introduction to population genetics theory.

[2] Kimura M (1983). The neutral theory of molecular evolution.

[3] Lu J, Tang T, Tang H, Huang J, Shi S, Wu CI (2006). The accumulation of deleterious mutations in rice genomes: a hypothesis on the cost of domestication. Trends Genet.

[4] Frantz LAF, Bradley DG, Larson G, Orlando L (2020). Animal domestication in the era of ancient genomics. Nat Rev Genet.

[5] Bosse M, Megens HJ, Derks MFL, de Cara AMR, Groenen MAM (2018). Deleterious alleles in the context of domestication, inbreeding, and selection. Evol Appl.

[6] Schubert M, Jonsson H, Chang D, Der Sarkissian C, Ermini L, Ginolhac A (2014). Prehistoric genomes reveal the genetic foundation and cost of horse domestication. Proc Natl Acad Sci USA.

[7] Marsden CD, Ortega-Del Vecchyo D, O'Brien DP, Taylor JF, Ramirez O, Vila C (2016). Bottlenecks and selective sweeps during domestication have increased deleterious genetic variation in dogs. Proc Natl Acad Sci USA.

[8] Makino T, Rubin CJ, Carneiro M, Axelsson E, Andersson L, Webster MT (2018). Elevated proportions of deleterious genetic variation in domestic animals and plants. Genome Biol Evol.

[9] Liu Q, Zhou Y, Morrell PL, Gaut BS (2017). Deleterious variants in Asian rice and the potential cost of domestication. Mol Biol Evol.

[10] Kono TJ, Fu F, Mohammadi M, Hoffman PJ, Liu C, Stupar RM (2016). The role of deleterious substitutions in crop genomes. Mol Biol Evol.

[11] Ramu P, Esuma W, Kawuki R, Rabbi IY, Egesi C, Bredeson JV (2017). Cassava haplotype map highlights fixation of deleterious mutations during clonal propagation. Nat Genet.

[12] Renaut S, Rieseberg LH (2015). The accumulation of deleterious mutations as a consequence of domestication and improvement in sunflowers and other compositae crops. Mol Biol Evol.

[13] Henn BM, Botigué LR, Peischl S, Dupanloup I, Lipatov M, Maples BK (2016). Distance from sub-Saharan Africa predicts mutational load in diverse human genomes. Proc Natl Acad Sci USA.

[14] Subramanian S (2016). Europeans have a higher proportion of highfrequency deleterious variants than Africans. Hum Genet.

[15] Xie X, Yang Y, Ren Q, Ding X, Bao P, Yan B (2018). Accumulation of deleterious mutations in the domestic yak genome. Anim Genet.

[16] Michot P, Chahory S, Marete A, Grohs C, Dagios D, Donzel E (2016). A reverse genetic approach identifies an ancestral frameshift mutation in RP1 causing recessive progressive retinal degeneration in European cattle breeds. Genet Sel Evol.

[17] Pitt D, Sevane N, Nicolazzi EL, MacHugh DE, Park SDE, Colli L (2018). Domestication of cattle: two or three events?. Evol Appl.

[18] Gibbs RA, Taylor JF, Van Tassell CP, Barendse W, Eversole KA, Bovine HapMap Consortium (2009). Genome-wide survey of SNP variation uncovers the genetic structure of cattle breeds. Science.

[19] Chen N, Fu W, Zhao J, Shen J, Chen Q, Zheng Z (2020). BGVD: An integrated database for bovine sequencing variations and selective signatures. Genomics Proteomics Bioinformatics.

[20] Yang Z (2007). PAML 4: phylogenetic analysis by maximum likelihood. Mol Biol Evol.

[21] Cooper GM, Stone EA, Asimenos G, Green ED, Batzoglou S, NISC Comparative Sequencing Program (2005). Distribution and intensity of constraint in mammalian genomic sequence. Genome Res.

[22] Fu W, O'Connor TD, Jun G, Kang HM, Abecasis G, Leal SM (2013). Analysis of 6515 exomes reveals the recent origin of most human protein-coding variants. Nature.

[23] Tennessen JA, Bigham AW, O'Connor TD, Fu W, Kenny EE, Gravel S (2012). Evolution and functional impact of rare coding variation from deep sequencing of human exomes. Science.

[24] Huber CD, Kim BY, Lohmueller KE (2020). Population genetic models of GERP scores suggest pervasive turnover of constrained sites across mammalian evolution. PLoS Genet.

[25] Lawrie DS, Petrov DA (2014). Comparative population genomics: power and principles for the inference of functionality. Trends Genet.

[26] Tajima F (1989). Statistical method for testing the neutral mutation hypothesis by DNA polymorphism. Genetics.

[27] Chen N, Cai Y, Chen Q, Li R, Wang K, Huang Y (2018). Whole-genome resequencing reveals world-wide ancestry and adaptive introgression events of domesticated cattle in East Asia. Nat Commun.

[28] Koenig D, Jimenez-Gomez JM, Kimura S, Fulop D, Chitwood DH, Headland LR (2013). Comparative transcriptomics reveals patterns of selection in domesticated and wild tomato. Proc Natl Acad Sci USA.

[29] Barbato M, Hailer F, Upadhyay M, Del Corvo M, Colli L, Negrini R (2020). Adaptive introgression from indicine cattle into white cattle breeds from Central Italy. Sci Rep.

[30] Barbato M, Reichel MP, Passamonti M, Low WY, Colli L, Tearle R (2020). A genetically unique Chinese cattle population shows evidence of common ancestry with wild species when analysed with a reduced ascertainment bias SNP panel. PLoS One.

[31] Chagunda MGG, Mujibi FDN, Dusingizimana T, Kamana O, Cheruiyot E, Mwai OA (2018). Use of high density single nucleotide polymorphism (SNP) arrays to assess genetic diversity and population structure of dairy cattle in smallholder dairy systems: The case of Girinka programme in Rwanda. Front Genet.

[32] Gebrehiwot NZ, Strucken EM, Aliloo H, Marshall K, Gibson JP (2020). The patterns of admixture, divergence, and ancestry of African cattle populations determined from genome-wide SNP data. BMC Genomics.

[33] Matukumalli LK, Lawley CT, Schnabel RD, Taylor JF, Allan MF, Heaton MP (2009). Development and characterization of a high density SNP genotyping assay for cattle. PLoS One.

[34] McTavish EJ, Hillis DM (2015). How do SNP ascertainment schemes and population demographics affect inferences about population history?. BMC Genomics.

[35] Hayes BJ, Daetwyler HD (2019). 1000 bull genomes project to map simple and complex genetic traits in cattle: applications and outcomes. Annu Rev Anim Biosci.

[36] Stafuzza NB, Zerlotini A, Lobo FP, Yamagishi ME, Chud TC, Caetano AR (2017). Single nucleotide variants and InDels identified from whole-genome re-sequencing of Guzerat, Gyr, Girolando and Holstein cattle breeds. PLoS One.

[37] Cai X, Chen H, Lei C, Wang S, Xue K, Zhang B (2007). mtDNA diversity and genetic lineages of eighteen cattle breeds from *Bos taurus* and *Bos indicus* in China. Genetica.

[38] Xia X, Huang G, Wang Z, Sun J, Wu Z, Chen N (2019). Mitogenome diversity and maternal origins of Guangxi cattle breeds. Animals (Basel).

[39] Xia X, Qu K, Zhang G, Jia Y, Ma Z, Zhao X (2019). Comprehensive analysis of the mitochondrial DNA diversity in Chinese cattle. Anim Genet.

[40] Xia XT, Achilli A, Lenstra JA, Tong B, Ma Y, Huang YZ (2021). Mitochondrial genomes from modern and ancient Turano-Mongolian cattle reveal an ancient diversity of taurine maternal lineages in East Asia. Heredity (Edinb).

[41] Achilli A, Bonfiglio S, Olivieri A, Malusà A, Pala M, Hooshiar Kashani B (2009). The multifaceted origin of taurine cattle reflected by the mitochondrial genome. PLoS One.

[42] Achilli A, Olivieri A, Pellecchia M, Uboldi C, Colli L, Al-Zahery N (2008). Mitochondrial genomes of extinct aurochs survive in domestic cattle. Curr Biol.

[43] Bollongino R, Burger J, Powell A, Mashkour M, Vigne JD, Thomas MG (2012). Modern taurine cattle descended from small number of near-eastern founders. Mol Biol Evol.

