# Supplementary material for: Deleterious protein-coding variants in diverse cattle breeds of the world
Source: Genet Sel Evol. 2021 Oct 15;53:80. doi: 10.1186/s12711-021-00674-7 (PMC8518297; doi:10.1186/s12711-021-00674-7)
Supplement: Supplementary file 1 — Additional file 1: Table S1. Locations and number of samples for each cattle breed. [file 12711_2021_674_MOESM1_ESM.docx]

**Table S1 Sample information**

| **Species** | **Breed** | **Sample #** | **Location** | **Region** |
| --- | --- | --- | --- | --- |
| *Bos taurus* | Angus | 25 | Aberdeenshire, Scotland, UK | West Europe |
| *Bos taurus* | RedAngus | 16 | Aberdeenshire, Scotland, UK | West Europe |
| *Bos taurus* | Hereford | 21 | Hereford, UK | West Europe |
| *Bos taurus* | Holstein | 45 | De Kooy, North Holland, Holland | West Europe |
| *Bos taurus* | Devon | 1 | Cork, UK | West Europe |
| *Bos taurus* | MaineAnjou | 6 | Lyon, France | Central_South_Europe |
| *Bos taurus* | Charolais | 14 | Charolais, France | Central_South_Europe |
| *Bos taurus* | Salers | 1 | Auvergne, Franch | Central_South_Europe |
| *Bos taurus* | Limousin | 1 | Limousin, France | Central_South_Europe |
| *Bos taurus* | Piedmontese | 5 | Piedmontese, Italy | Central_South_Europe |
| *Bos taurus* | Gelbvieh | 21 | München, Bavaria, Germany | Central_South_Europe |
| *Bos taurus* | Simmental | 23 | Geneve, Switzerland | Central_South_Europe |
| *Bos taurus* | Jersey | 12 | Channel Island of Jersey, UK | Central_South_Europe |
| *Bos taurus* $\times$ *Bos indicus* | Rashoki | 9 | Sanandaj, Iran | Middle_East |
| *Bos taurus* | Kazakh | 9 | Yining,Yili, Xinjing, China | Northwest_China |
| *Bos taurus* | Mongolian | 7 | Kuerl, Bayingolin, Xinjiang, China | Northwest_China |
| *Bos taurus* | Chaidamu | 5 | Geermu, Haixi, Qinghai, China | Northwest_China |
| *Bos taurus* | Tibetan | 9 | Changdu,Xizang, China | Tibet |
| *Bos taurus* | Yanbian | 1 | Yanbian, Jilin, China | Northeast_Asia |
| *Bos taurus* | Hanwoo | 17 | Chuncheon, Kangwon-do, Korea | Northeast_Asia |
| *Bos taurus* | Kuchinoshima | 1 | Kuchinoshima, Japan | Northeast_Asia |
| *Bos taurus* | Mishima | 8 | Mishima Island, Japan | Northeast_Asia |
| *Bos taurus* $\times$ *Bos indicus* | BohaiBlack | 5 | Binzhou, Shandong, China | North-Central China |
| *Bos taurus* $\times$ *Bos indicus* | Luxi | 5 | Jining, Shandong, China | North-Central China |
| *Bos taurus* $\times$ *Bos indicus* | JiaxianRed | 5 | Jiaxian, Pingdingshan, Henan, China | North-Central China |
| *Bos taurus* $\times$ *Bos indicus* | Nanyang | 5 | Nanyang, Henan, China | North-Central China |
| *Bos taurus* $\times$ *Bos indicus* | Bashan | 5 | Xuanhan, Dazhou, Sichuan, China | North-Central China |
| *Bos taurus* $\times$ *Bos indicus* | Lingnan | 8 | Shangluo, Shanxi, China | North-Central China |
| *Bos indicus* | Wandong | 2 | Jixi, Anhui, China | North-Central China |
| *Bos taurus* $\times$ *Bos indicus* | Zaobei | 5 | Zaoyang, Xiangyang, Hubei, China | North-Central China |
| *Bos taurus* $\times$ *Bos indicus* | Dabieshan | 2 | Weining, Bijie, Guizhou, China | North-Central China |
| *Bos taurus* $\times$ *Bos indicus* | Weining | 5 | Weining, Bijie, Guizhou, China | North-Central China |
| *Bos indicus* | Wannan | 5 | Jixi, Anhui, China | South_China |
| *Bos indicus* | Jinjiang | 3 | Gaoan, Yichuan, Jiangxi, China | South_China |
| *Bos indicus* | Guangfeng | 4 | Guangfeng, Shangrao, Jiangxi, China | South_China |
| *Bos indicus* | Jian | 4 | Ji’an, Jiangxi, China | South_China |
| *Bos indicus* | Leiqiong | 3 | Leizhou, Guangdong,China | South_China |
| *Bos indicus* | Wenshan | 8 | Guangnan,Wenshan,China | South_China |
| *Bos indicus* | Dianzhong | 6 | Jiangcheng, Puer,Yunnan,China | South_China |
| *Bos indicus* | Gir | 3 | Amblash, Gujarat, India | India_Pakistan |
| *Bos indicus* | Nelore | 4 | Nelore, Andhra, India | India_Pakistan |
| *Bos indicus* | Brahman | 9 | Brahman, West Bengal, India | India_Pakistan |
| *Bos indicus* | Hariana | 1 | Gurgaon, Haryana, India | India_Pakistan |
| *Bos indicus* | Sahiwal | 1 | Sahiwal, Punjab, Pakistan | India_Pakistan |
| *Bos indicus* | Tharparkar | 1 | Tharparkar, Sindh, Pakistan | India_Pakistan |
| *Bos indicus* | Srilanka | 5 | Sirlank | India_Pakistan |
| *Bos taurus* $\times$ *Bos indicus* | ShorthornZebu | 10 | Mubende, Uganda (Closest city) | Africa |
| *Bos taurus* $\times$ *Bos indicus* | Nsongora | 1 | Kasese, Uganda (Closest city) | Africa |
| *Bos taurus* $\times$ *Bos indicus* | Ankole | 20 | Masindi, Uganda (Closest city) | Africa |
| *Bos taurus* $\times$ *Bos indicus* | Nganda | 1 | Masaka, Uganda (Closest city) | Africa |
| *Bos taurus* $\times$ *Bos indicus* | Ogaden | 9 | Ethiopia, Africa | Africa |
| *Bos taurus* $\times$ *Bos indicus* | Boran | 10 | Kenya, Africa | Africa |
| *Bos taurus* | NDama | 10 | Guinea, Africa | Africa |
| *Bos taurus* $\times$ *Bos indicus* | Kenana | 9 | Sudan, Africa | Africa |

#: number
